# Hyperkalemia and acute kidney failure associated with preoperative uterine artery embolization for a large uterine fibroid: a case report

**DOI:** 10.1186/s13256-016-1092-3

**Published:** 2016-11-01

**Authors:** Keiko Tanaka, Toshimitsu Koizumi, Takeru Higa, Noriaki Imai

**Affiliations:** Hachinohe City Hospital, Hachinohe City, Japan

**Keywords:** Uterine artery embolization, Uterine fibrosis, Complications, Hyperkalemia, Acute kidney failure

## Abstract

**Background:**

Preoperative uterine artery embolization has been shown to help reduce blood loss, with few complications. Most reports indicated that uterine artery embolization is safe for uterine fibrosis; the occurrence of hyperkalemia and acute kidney failure as complications of preoperative uterine artery embolization has not been reported previously. Here we report the occurrence of hyperkalemia and acute kidney failure after preoperative uterine artery embolization for a large uterine fibroid. To the best of our knowledge, this is the first report on the occurrence of hyperkalemia and acute kidney failure after preoperative uterine artery embolization.

**Case presentation:**

A 48-year-old Japanese woman presented to our hospital complaining of compression in her abdomen and an abdominal mass. Magnetic resonance imaging showed a large uterine fibroid measuring 37.5×27×13.5 cm. Therefore, we planned preoperative uterine artery embolization to help reduce blood loss. However, hyperkalemia and acute kidney failure occurred owing to the development of necrotic tissue after uterine artery embolization; therefore, emergency total abdominal hysterectomy and bilateral salpingo-oophorectomy were performed. She experienced 105 g of blood loss intraoperatively. The weight of her uterus was 10.8 kg and the volume was 9964 cm^3^, with extensive necrotic tissue. Her hyperkalemia and kidney failure resolved after the surgery.

**Conclusions:**

We reported the occurrence of serious complications, including hyperkalemia and acute kidney failure, after preoperative uterine artery embolization for a large uterine fibroid.

## Background

Uterine artery embolization (UAE) for the treatment of uterine fibroids was first reported in 1995 [1], and since then, many reports [2–15] have described the risks and benefits of UAE. Some reports [12–15] stated that preoperative UAE can help reduce bleeding, and most reports indicated that UAE is safe for uterine fibroid. David et al. [15] reported that patients undergoing a hysterectomy with a uterine weight of more than 1000 g have a significantly higher risk of perioperative complications and are at greater risk of requiring a blood transfusion. David and Kröncke [15] also reported that only two of the three patients with myomata weighing more than 1100 g were able to avoid blood transfusion, because of preoperative UAE. The occurrence of hyperkalemia and acute kidney failure as complications of preoperative UAE has not been reported previously. Here we report the occurrence of hyperkalemia and acute kidney failure after preoperative UAE for a large uterine fibroid. To our knowledge, this is the first report on the occurrence of hyperkalemia and acute kidney failure after preoperative UAE.

## Case presentation

**Fig. 1:**
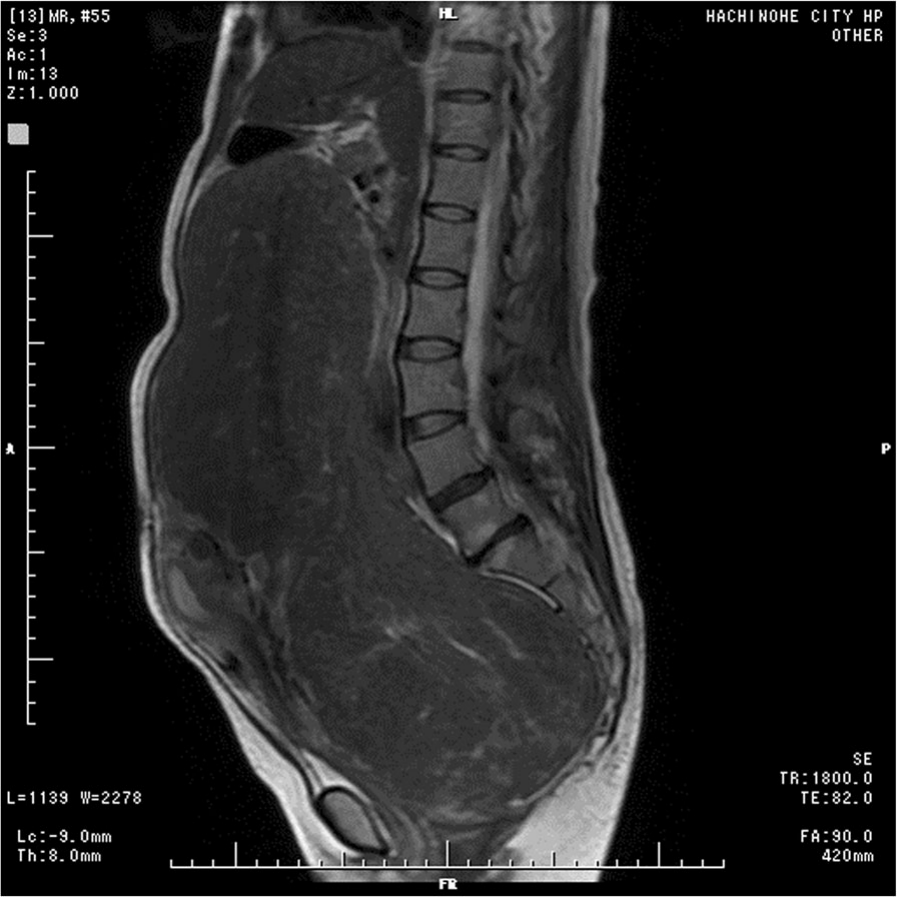
A sagittal T2-weighted magnetic resonance image of the uterus

**Fig. 2:**
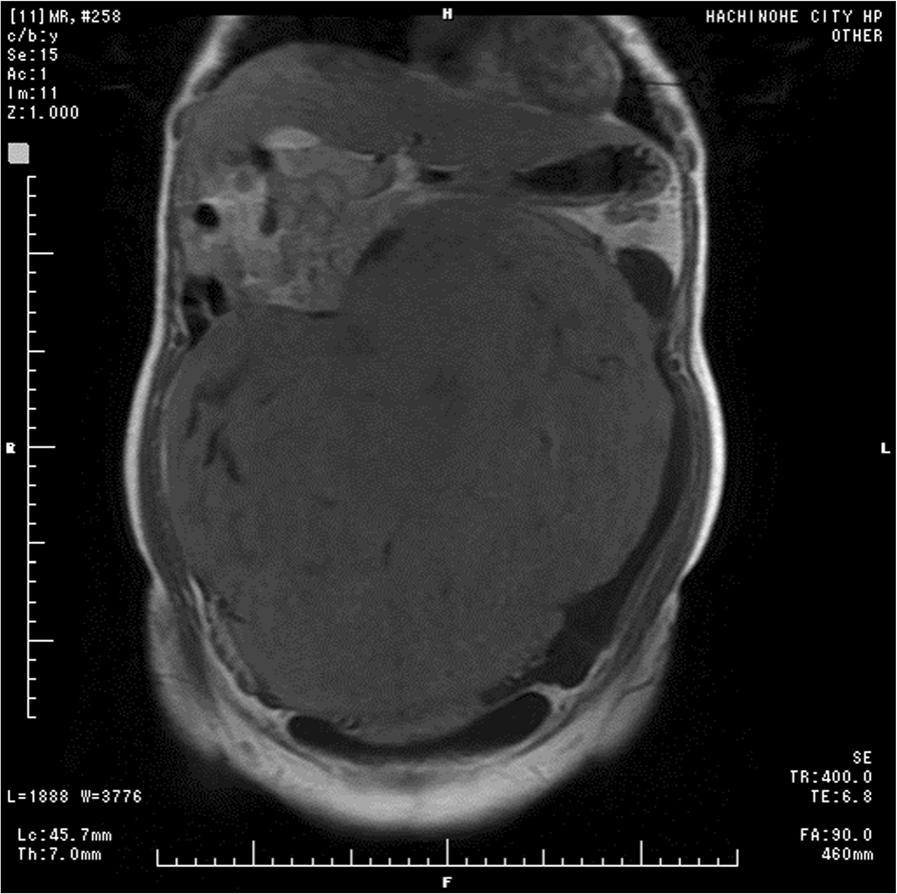
A coronal T2-weighted magnetic resonance image of the uterus

A 48-year-old Japanese woman with a medical history of multiple sclerosis presented to our hospital complaining of compression in her abdomen and an abdominal mass. Magnetic resonance imaging revealed a uterine fibroid measuring 37.5×27×13.5 cm along with some small fibroids (Figs. 1 and 2). We planned total abdominal hysterectomy and bilateral salpingo-oophorectomy 3 days after UAE.

**Fig. 3:**
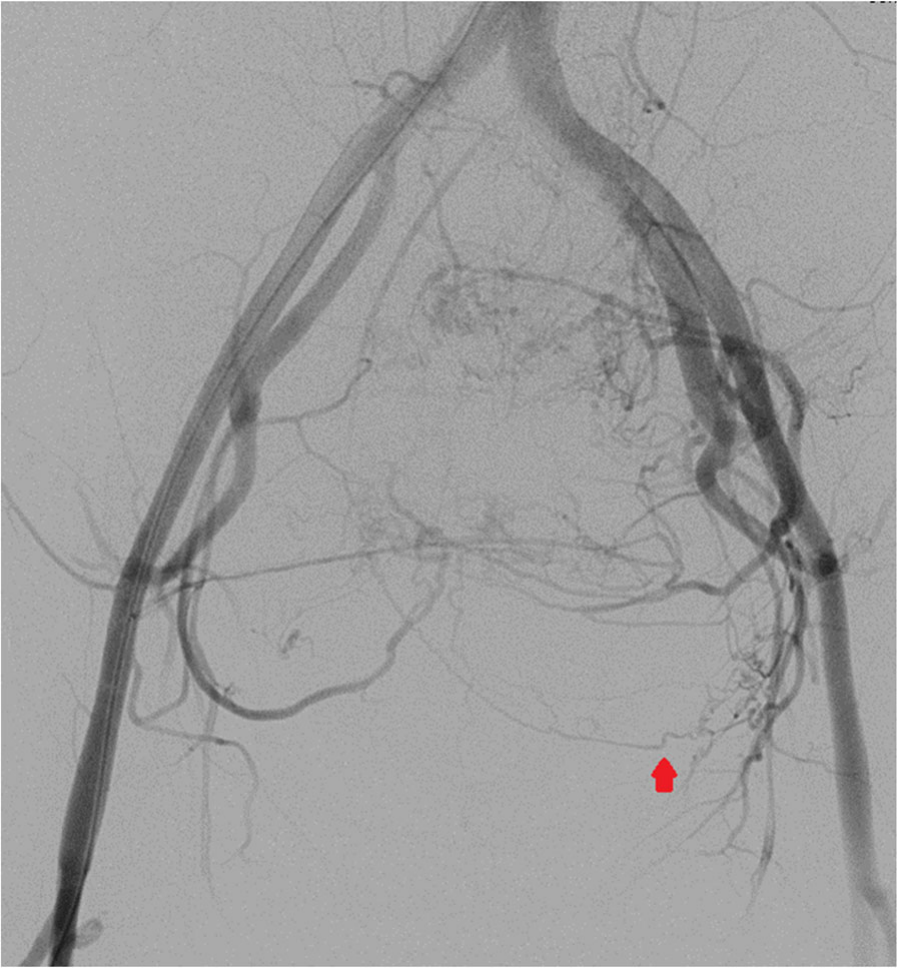
An angiography image obtained before uterine artery embolization. The left bladder artery (*arrow*) supplied a uterine fibroid

**Fig. 4:**
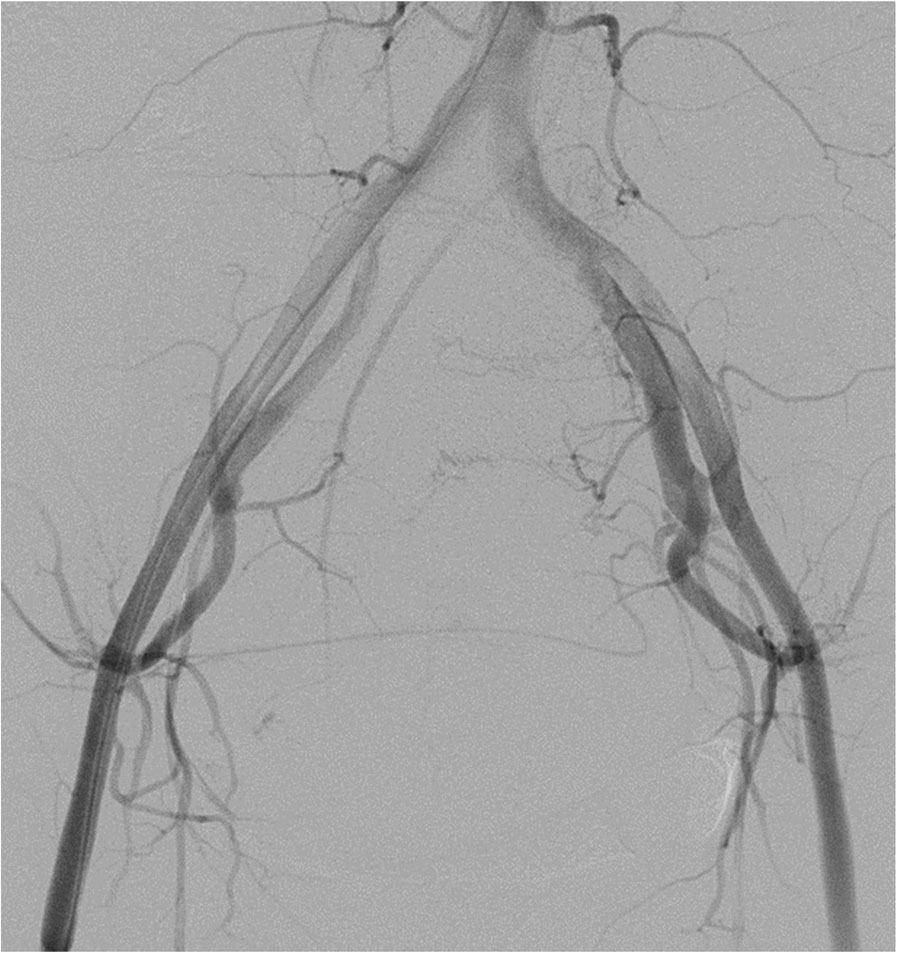
An angiography image obtained after uterine artery embolization

**Fig. 5:**
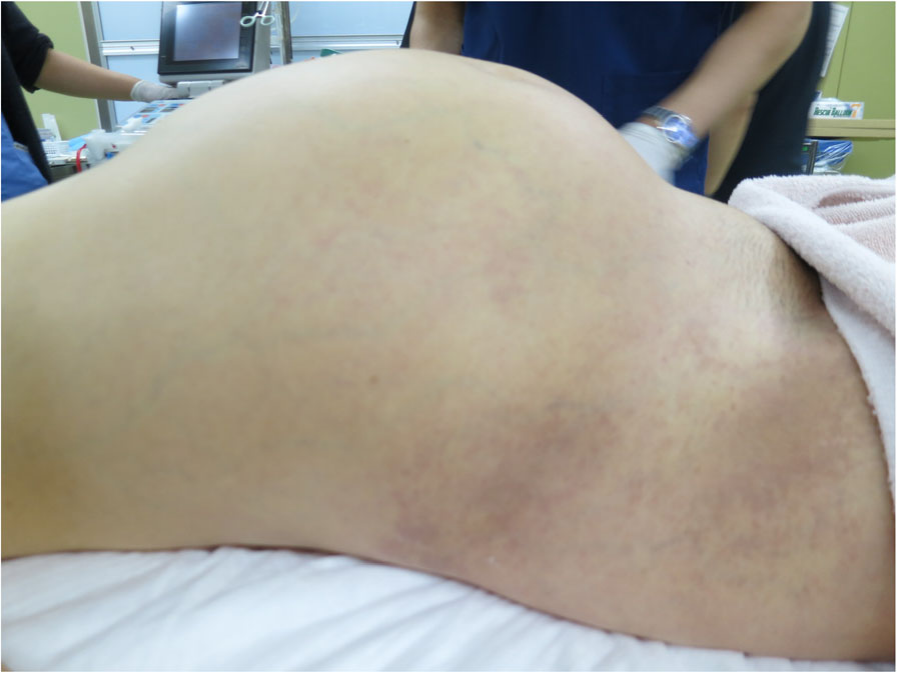
An image of the patient’s abdomen before surgery

**Fig. 6:**
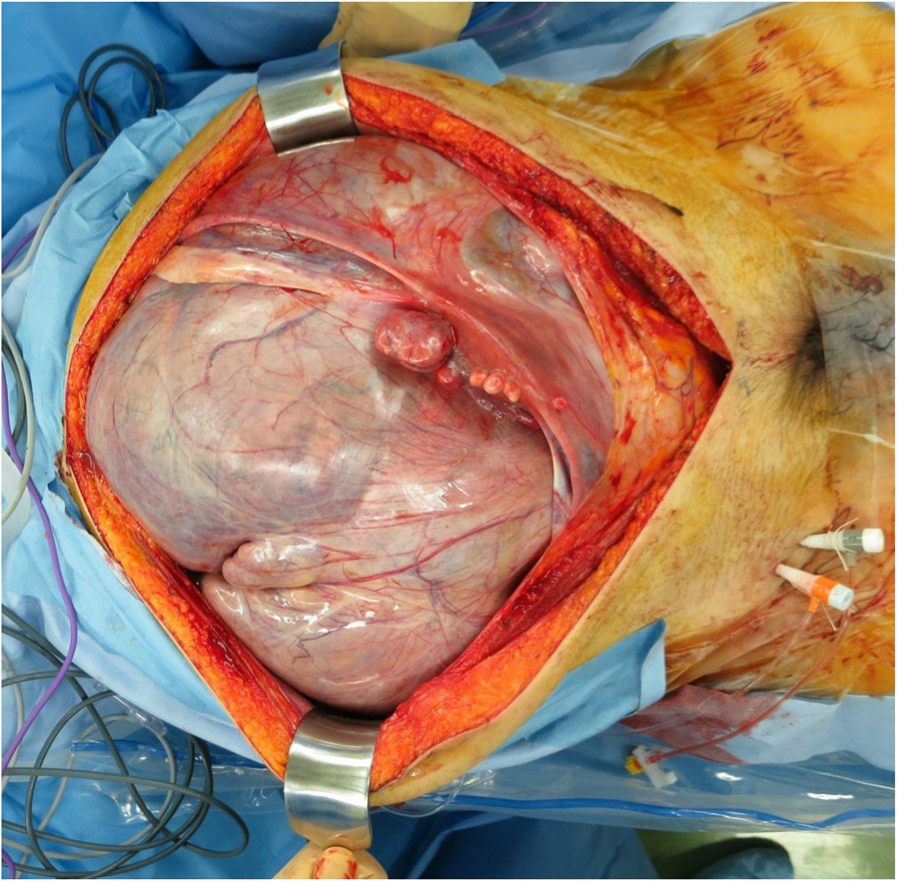
An image of the abdomen obtained during the surgery. The uterus occupies a large part of the intraperitoneal space

**Fig. 7:**
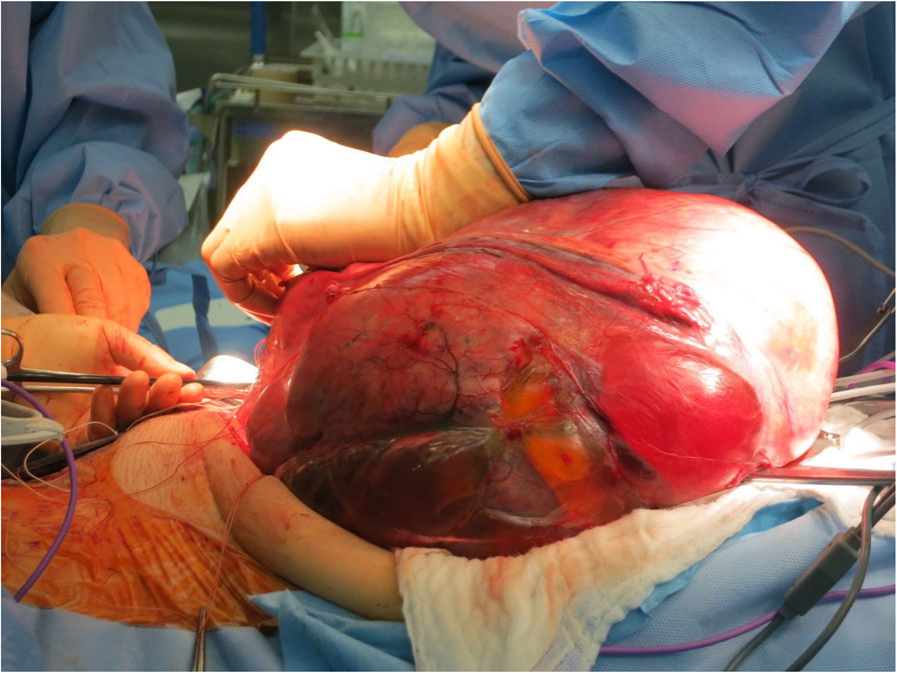
An image of the uterus during the surgery

**Fig. 8:**
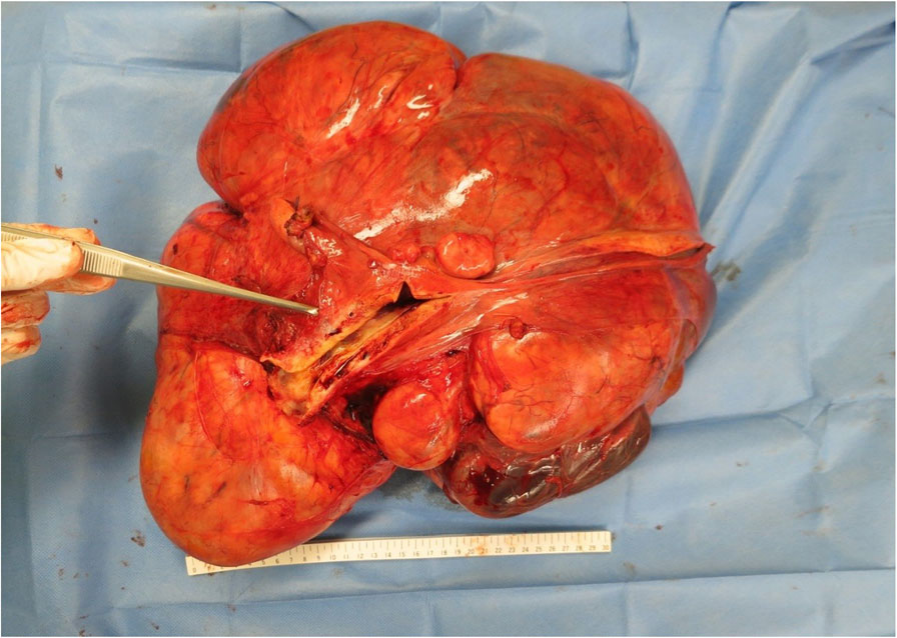
An image of the excised specimen

**Fig. 9:**
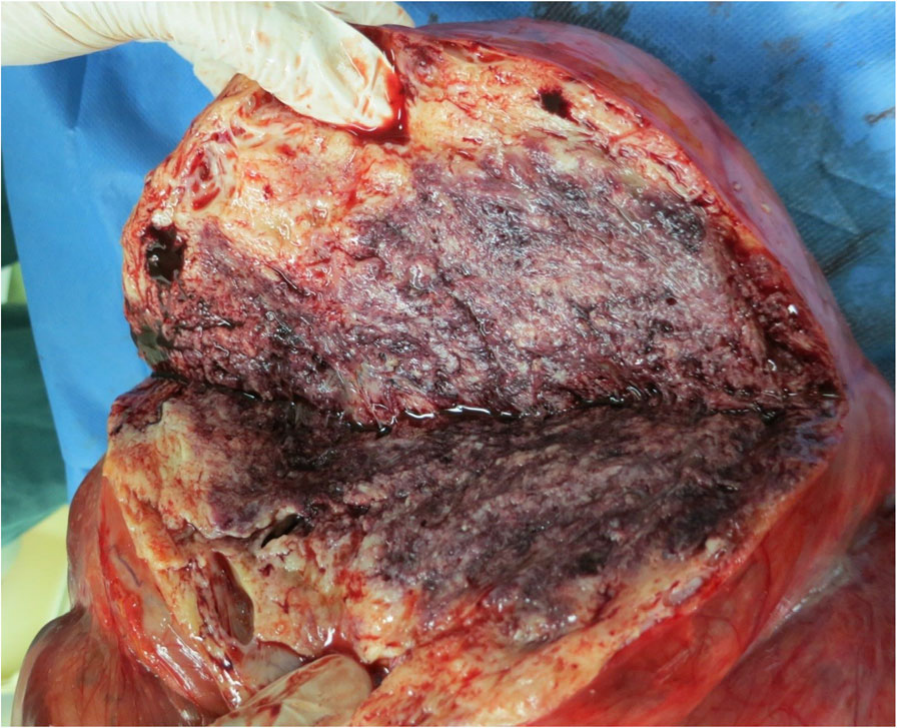
An image showing necrosis within the excised specimen

**Fig. 10:**
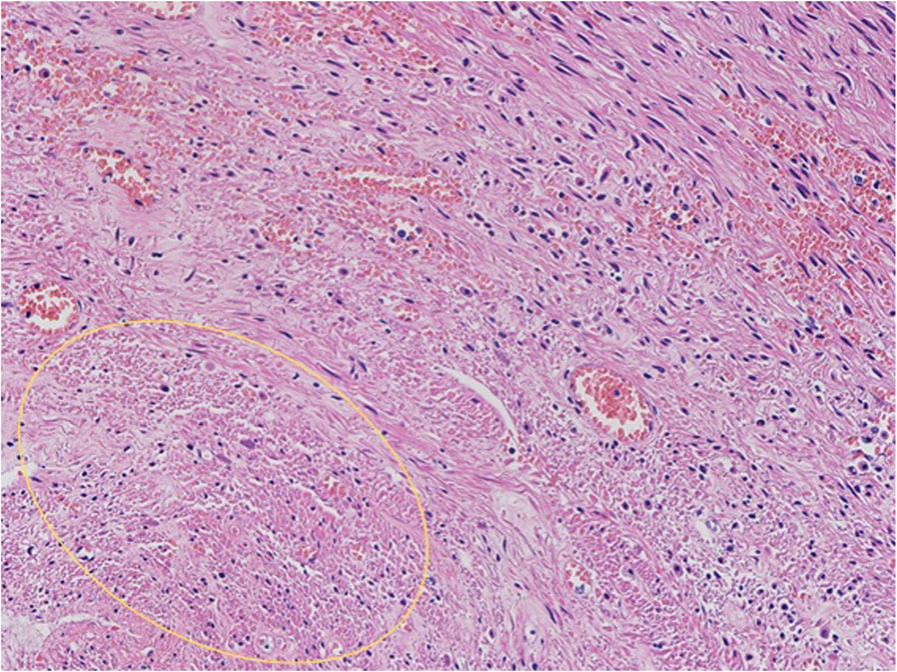
A histological image showing necrotic cells (*yellow circle*). Hematoxylin and eosin stain, ×400

Embolization of her bilateral uterine arteries and selective embolization of her left bladder artery were performed using a gelatin sponge (Figs. 3 and 4), because her left bladder artery (Fig. 3, arrow) supplied the uterine fibroid. However, 12 hours after embolization, she experienced cold sweats and vomiting, and 15 hours after embolization, hyperkalemia was noted on venous blood analysis and acute kidney failure was identified (Table 1). Arterial blood gas analysis showed compensated metabolic acidosis: pH, 7.368; partial pressure of carbon dioxide (pCO_2_), 27.3 mmHg; base excess, −8.2; and bicarbonate (HCO_3_), 15.4 mmol/L. Glucose-insulin therapy was administered; however, it was not successful in resolving her condition. She then received continuous hemodiafiltration in our intensive care unit; however, her hyperkalemia and kidney failure did not improve. Therefore, she underwent emergency surgery. Total abdominal hysterectomy and bilateral salpingo-oophorectomy were performed, and her intraoperative blood loss was 105 g (Figs. 5, 6, and 7). The weight of her uterus was 10.8 kg and the volume was 9964 cm^3^. The volume was calculated using the formula: volume = length (cm) × width (cm) × diameter (cm) × 0.5233 (Fig. 8). She underwent autotransfusion (800 mL) and received 1200 mL of packed red blood cells. Her uterus had necrotic tissue, and the pathological finding was uterine fibrosis with necrosis (Figs. 9 and 10). Following surgery, her hyperkalemia and kidney failure resolved.

**Table 1:** Results of venous blood analysis 15 hours after uterine artery embolization

Test	Value
White blood cell count (μL)	24800
Red blood cell count (×10^4^/μL)	454
Hemoglobin (g/dL)	13.3
Hematocrit (%)	39.3
Platelet count (×10^4^/μL)	32.5
Total protein (g/dL)	9.4
Albumin (g/dL)	4.7
Potassium (mEq/L)	8.0
Sodium (mEq/L)	134
Chloride (mEq/L)	96
Blood urea nitrogen (mg/dL)	28
Creatinine (mg/dL)	2.02
Creatine kinase (IU/L)	301
Lactate dehydrogenase (IU/L)	363
Aspartate transaminase (IU/L)	33
Alanine transaminase (IU/L)	17
Alkaline phosphatase (IU/L)	262
C-reactive protein (mg/dL)	1.49
Activated partial thromboplastin time (s)	25.6
Prothrombin time international normalized ratio	1.04
Fibrinogen (mg/dL)	452

## Discussion

Previous reports [2–15] mentioned that most women who underwent UAE for uterine fibroids were satisfied with the clinical outcome and had few complications. Although complications have been reported after UAE, hyperkalemia and kidney failure have not been reported previously.

The complication rate associated with UAE has been reported to be very low, and most complications have been found to be transient [2]. The most serious complication associated with UAE is endometritis/uterine infection, with a reported incidence of approximately 2 % [2–4]; however, the associated morbidity rate was found to be very low [2]. Complications following UAE can be classified into immediate (peri-procedure), early (within 30 days), and late (beyond 30 days) complications [5]. Most immediate complications are local complications, such as hematoma, arterial thrombosis, dissection, and pseudoaneurysm, and other complications include spasm and non-target embolization [5]. Non-target embolization is relatively rare, and it does not occur if a good technique is used [5]. Most early complications are associated with post-embolization syndrome and include pain, nausea, fever, and malaise, and other complications are rare [5]. Most complications of UAE have been shown to occur more than 30 days after the procedure [5]. Late complications include vaginal discharge, fibroid expulsion and impaction, infection, amenorrhea, and sexual dysfunction [5]. The rate of hysterectomy subsequent to UAE ranges from 0.25 to 1.6 % [2–4]. Uterine necrosis is a rare complication after UAE, and it necessitates hysterectomy and treatment with antibiotics to prevent bacteremia, sepsis, and death [2, 6]. In our case, we believe that the uterine fibroids became necrotic following UAE and the necrotic tissue caused hyperkalemia and acute kidney failure. Some reports have mentioned that patients with a septic uterus required urgent surgery 7 or more days after the initial procedure [2, 6]. However, in our case, necrotic tissue caused serious complications and necessitated surgical intervention within 48 hours. In addition, our patient needed a blood transfusion in spite of the small intraoperative blood loss; we think a blood transfusion was needed because after UAE the necrotic uterine fibroids lost blood.

Previous reports found that the size of the uterine fibroid was not associated with complications after UAE [7–10]. In one report, complications were found to be associated with a large uterus size (500 mL), large dominant tumor volume (100 cm^3^), and high post-intervention creatine kinase level (170 U/L) [11]. Among previous reports, the largest fibroid was approximately 4000 cm^3^ [7–10], while the uterus in the present case was 9964 cm^3^; therefore, it was not possible to generalize the previous findings to the present case. In our case, fibroid volume was the most important risk factor for serious complications.

It has been reported that surgical intervention should be performed within 48 hours after preoperative UAE [12–15]. Therefore, for the prevention of serious complications, such as those in the present case, we suggest that surgical intervention should be performed immediately after preoperative UAE.

## Conclusions

We reported the occurrence of serious complications, including hyperkalemia and acute kidney failure, after preoperative UAE for a large uterine fibroid. Preoperative UAE is effective for preventing blood loss. The findings of the present case indicate that UAE performed for a large uterine fibroid can cause hyperkalemia and acute kidney failure.
